# Recurrent urinary tract infection associated with SGLT‐2 inhibitor in type 2 diabetes mellitus patient: A case report

**DOI:** 10.1002/ccr3.6803

**Published:** 2023-01-03

**Authors:** Mohammad Hazique, Arihant Surana, Mehul Sinha, Ayush Anand

**Affiliations:** ^1^ Nilratan Sircar Medical College and Hospital Kolkata India; ^2^ Kasturba Medical College Manglore India; ^3^ B. P. Koirala Institute of Health Sciences Dharan Nepal

**Keywords:** case report, diabetes mellitus, empagliflozin, urinary tract infection

## Abstract

A high index of clinical suspicion of urinary tract infections due to empagliflozin use should be maintained in T2DM patients to avoid progression to life‐threatening condition.

## 1 INTRODUCTION

Type 2 diabetes mellitus (T2DM) is a chronic disease in which the body is unable to adequately use the insulin produced in the pancreas.^1^ Type 2 diabetes mellitus leads to various microvascular and macrovascular complications such as neuropathy, retinopathy, cerebrovascular disease, kidney failure, and coronary heart disease and is associated with an increased risk of infections.^2^ A meta‐analysis by Salari et al.^3^ found the prevalence of urinary tract infections (UTI) in T2DM patients to be 11.5% approximately. People with diabetes are particularly susceptible to UTIs due to immune dysfunction and increased adherence of bacteria to the urothelium.^4^ In addition, oral hypoglycemic drugs such as sodium‐glucose co‐transporter 2 inhibitors (SGLT‐2i) leading to glycosuria can further increase the risk of developing UTI.^4^ However, except for dapagliflozin, no significant association between SGLT‐2i and UTI was found.^5^ Despite this, the development of UTIs following SGLT2i can not be ruled out due to wider confidence intervals reported in studies.^5^ Here, we report a case of UTI following empagliflozin use in a diabetic female patient.

## 2 CASE REPORT

A 39‐year‐old female patient with T2DM for the past 15 years presented to OPD with flank pain, fever, and malaise for 1 week. Her past history revealed that she was managed with Metformin 1000 mg and Glimepiride 2 mg daily. Ten months back, she visited another hospital for follow‐up, and due to poor control of HbA1c (increased to 7.6% from 6.8% over 2 months), empagliflozin 10 mg was added. After 2 months of starting empagliflozin, she had multiple episodes of increased frequency and urgency of urination, along with burning micturition for the next 8 months, which were self‐limiting. During these episodes, she did not experience any fever, chills, nausea, vomiting, hematuria, or lower abdominal discomfort.

**FIGURE 1:**
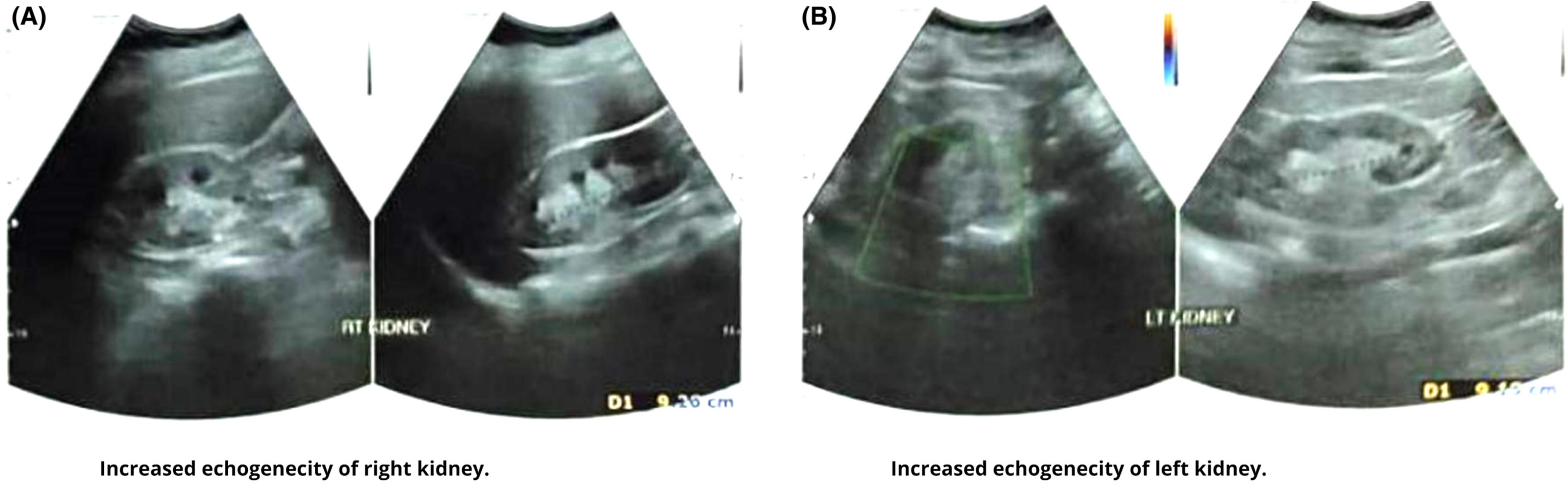
USG showing increased echogenicity of bilateral kidneys

Her vitals were blood pressure of 128/70 mm of Hg, pulse rate of 80 beats per minute, the temperature of 38.4°C, and Spo2 was 97%. On physical examination, she had a BMI of 24.2 kg/m^2^ with costovertebral angle tenderness, dry mucous membranes, and decreased skin turgor. The laboratory investigations (Table 1) revealed the growth of Escherichia coli and antibiotic sensitivity to cefixime, nitrofurantoin, and ceftriaxone; her blood culture was negative. Urologist consultation was performed, and based on clinical evaluation and laboratory investigations, a provisional diagnosis of UTI was made, and ultrasonography (USG) of the abdomen and pelvis was done. The USG (Figure 1) revealed increased echogenicity of bilateral kidneys (Bilateral Renal Parenchymal Disease Grade 1) with postvoid urine measuring 100 ml.

**TABLE 1:** Laboratory investigations on OPD visit

Investigations	Results	Reference range
CBC (cells/μl)	8700	4000–10,000
DLC (%)	N 74.4%	N 40–70
Hb (g/dl)	11	12–15
Platelets (cells/μl)	226,000	150,000–450,000
BUN (mg/dl)	38	10–40
Serum creatinine (mg/dl)	1.5	0.52–1.04
UACR (mg/g)	88.08	<30
FBG (mg/dl)	237	
PPBG (mg/dl)	282	
HbA1c (%)	8.2	
Urinalysis		
Specific gravity	1.010	<1.005
Occult blood	Positive (1+)	Absent
Leucocyte esterase	Positive	Negative
Protein	Positive (+2)	Negative
Glucose	Positive (+2)	Negative
Nitrites	Positive	Negative
RBCs (per hpf)	3–4	Negative
WBC (per hpf)	30–40	0–5
Casts	Nil	Absent
Crystals	Negative	Absent
Urine culture	Escherichia coli (>100,000/mm^3^)	<100,000/mm^3^
Antibiotic sensitivity	Sensitive to cefixime, nitrofurantoin, and ceftriaxone	

Abbreviations: BUN, blood urea nitrogen; CBC, complete blood cell count; DLC, differential leucocyte count; FBG, fasting blood glucose; Hb, hemoglobin; HbA1c, glycated hemoglobin; N, neutrophils; PPBG, 2‐hour postprandial glucose; RBC, red blood cell; UACR, urine albumin‐to‐creatinine ratio; WBC, white blood cell.

She was admitted to the hospital and started on intravenous Ceftriaxone 1 g once daily and oral Azithromycin 500 mg twice daily for 5 days. She was afebrile after 24 h of starting the medications, and the rest of her symptoms improved. The rest of her hospital stay was uneventful, and she was discharged after 6 days. On discharge, she was started on Metformin extended‐release (ER) oral tablets 500 mg daily, Linagliptin/Metformin ER 5 mg/500 mg oral daily, oral empagliflozin 10 mg once daily, and oral tamsulosin 0.4 mg once daily. She was also advised to take oral cefixime twice daily for the next 5 days, with a follow‐up in 3 weeks.

At her follow‐up visit 3 weeks later, she reported no symptoms. Her urinalysis and urine culture reports were both normal at this visit. Her blood test showed serum urea of 38 mg/dl, serum creatinine of 1.5 mg/dl, fasting blood sugar of 120 mg/dl, and 2‐hour postprandial blood sugar of 185 mg/dl.

**FIGURE 2:**
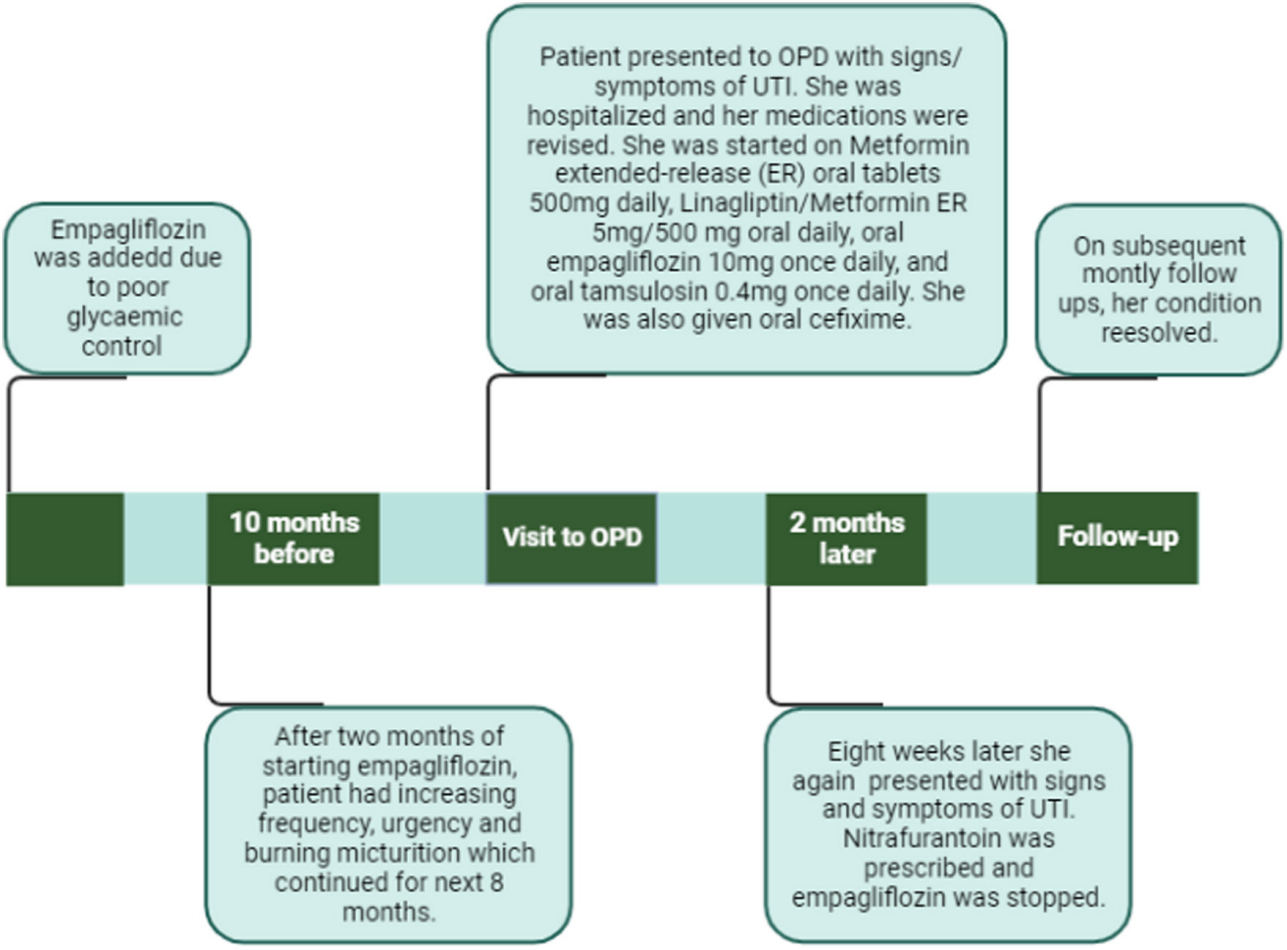
Time line of events (Original Image created with https://biorender.com). UTI, urinary tract infection

After 1 month, she reported increased urine frequency, urgency, and burning micturition. No fever, chills, nausea, vomiting, hematuria, or lower abdominal discomfort were noted. Her urinalysis revealed +1 for glucose and protein, with the presence of pus cells 10–20/hpf. Based on the previous sensitivity report (Table 1), Nitrofurantoin 100 mg for 5 days was prescribed, continuing with the rest of her medications, and empagliflozin was discontinued. Her symptoms gradually improved, and on subsequent monthly follow‐ups, she did not report urinary complaints after the stoppage of empagliflozin. Her urinalysis showed 5–6 pus cells/hpf on the first monthly follow‐up. The time line of events is described in Figure 2.

## 3 DISCUSSION

The diagnosis of UTI is made by clinical correlation of the presenting signs/symptoms with urinalysis or urine culture.^6^ Usually, the patients with lower UTI are asymptomatic; symptoms, if present, include dysuria, frequency, urgency, hematuria, and suprapubic pain.^4^, ^7^ The most commonly reported causative organism for UTI is E. Coli, which is more common in women and is a recurrent disease because of anatomically shorter urethra present in women.^6^, ^8^ Sometimes, the patients can develop recurrent UTIs, defined as two or more episodes of UTI in the last 6 months or three or more in the past 12 months.^9^ In our case, the initial urine culture report showed growth of E. coli. Based on clinical evaluation and urine culture, the diagnosis of recurrent UTI was made.

Hirji et al.^10^ found that women (adjusted RR = 1.53) were at higher risk of developing UTI than men (adjusted RR = 1.49). Studies have shown that recurrent UTI has a higher prevalence in T2DM,^11^ particularly in women taking oral hypoglycemic drugs (OR 2.1; 95% CI 1.2–3.5) or with diabetes ≥5 years (OR 2.9; 95% CI 1.9–4.4).^12^ In our case, the patient had risk factors such as female gender, T2DM, and oral hypoglycemic medications, which might have caused her to develop a UTI. However, there were no documented reports of UTI in the patient earlier to the recent presentation.

The proximal convoluted tubule in the kidney has sodium‐glucose co‐transporter 2 channels, increasing sodium and glucose reabsorption.^13^ Sodium‐glucose co‐transporter 2 inhibitors inhibits the sodium‐glucose co‐transporter 2 in the proximal convoluted tubule in the kidney. It leads to glycosuria, which may increase the risk of UTIs.^4^ Although SGLT2i are safe drugs for the management of T2DM,^14^ increased risk of various adverse effects such as diabetic ketoacidosis, genital infection, and urinary tract infection have been found.^15^ Various studies have reported an increased occurrence of UTIs following SGLT2i use. Hall et al.^16^ reported a case of UTI in a 70 year man with T2DM following dapagliflozin use. Brock et al.^17^ reported lower urinary tract infection case in a T2DM patient 4 months after initiation of empagliflozin. Moreover, US FDA adverse reporting database showed 19 cases of SGLT2i use in T2DM, where UTI preceded the development of urosepsis and pyelonephritis.^18^ Another drug used in our case is Metformin, which shows protective action against UTIs by upregulating the action of AMPs in the lysosomes of uroepithelial cells, leading to improved host defense and efficient killing of pathogens within or outside the uroepithelial cells.^19^ Furthermore, other drugs such as linagliptin, glimepiride, and tamsulosin are not associated with an increased risk of UTI.^20^, ^21^, ^22^ Also, after discontinuation of empagliflozin, followed by adequate management, the patient's condition improved, and she did not show any signs/symptoms on subsequent follow‐ups. Like our case, Hall et al.^16^ reported a case where UTI was resolved following discontinuation of SGLT2i use. These pieces of evidence add weight to our case as the patient developed UTI after 2 months of starting empagliflozin. Hence, the recurrent UTIs in our patients can be attributed to the use of SGLT2i.

For the management of SGLT2i‐associated UTI, discontinuation of the drug with adequate management is vital to prevent life‐threatening conditions.^18^ Antibiotics can be prescribed based on antibiotic susceptibility tests and the severity of the disease.^23^ Also, prophylactic antibiotic treatment with trimethoprim and nitrofurantoin is recommended in case of recurrent UTI.^9^ Besides pharmacological management, it is crucial to ensure adequate glycemic control in diabetes mellitus patients using medical nutrition therapy, exercise, and patient education, and encourage patients to drink sufficient water and empty the bladder entirely during voiding.^23^

## 4 CONCLUSION

We presented a 39‐year‐old female patient with T2DM for the past 15 years who developed recurrent UTI following SGLT2i use. On discontinuation of empagliflozin and adequate management, her condition improved. We highlighted the development of UTIs due to empagliflozin use. Also, we showed that other drugs used in patient management were not associated with an increased risk of UTIs.

## AUTHOR CONTRIBUTIONS

**Mohammad Hazique:** Conceptualization; data curation; formal analysis; investigation; methodology; project administration; resources; validation; visualization; writing – original draft; writing – review and editing. **Arihant Surana:** Conceptualization; data curation; formal analysis; investigation; methodology; project administration; resources; validation; visualization; writing – original draft; writing – review and editing. **Mehul Sinha:** Conceptualization; data curation; formal analysis; investigation; validation; writing – original draft; writing – review and editing. **Ayush Anand:** Conceptualization; data curation; formal analysis; investigation; methodology; project administration; supervision; validation; visualization; writing – original draft; writing – review and editing.

## FUNDING INFORMATION

No funding was received.

## CONFLICT OF INTEREST

No conflict of interest.

## ETHICAL APPROVAL

Ethical approval was not required.

## CONSENT

Written informed consent was obtained from the patient's legal guardian to publish this report in accordance with the journal's patient consent policy.

## GUARANTOR

MH is the Guarantor.

## Data Availability

This manuscript has all the data relevant to this case report included.
